# Idiopathic Acquired Hemophilia A With High-Titer Factor VIII Inhibitor in an Elderly Patient: A Case Report

**DOI:** 10.7759/cureus.108854

**Published:** 2026-05-14

**Authors:** Andrea Morris, Anid Hassan, Tariq Alnsour, Nabahat Shafi, Paula Gonzalez Espinosa, Michael Levitt

**Affiliations:** 1 Department of Internal Medicine, Hackensack Meridian Health Jersey Shore University Medical Center, Neptune, USA; 2 Department of Internal Medicine, Jersey Shore University Medical Center, Neptune, USA; 3 Department of Medicine, Dow University of Health Sciences, Civil Hospital Karachi, Karachi, PAK

**Keywords:** acquired hemophilia a, autoantibodies, bethesda assay, coagulopathy, factor viii inhibitor, immunosuppression, mixing study, prednisone, prolonged aptt, rituximab

## Abstract

Acquired hemophilia A is a rare autoimmune bleeding disorder caused by neutralizing autoantibodies against factor VIII. It typically affects older adults and may present with spontaneous mucocutaneous bleeding, extensive ecchymoses, soft-tissue hematomas, and isolated prolongation of the activated partial thromboplastin time. We report the case of an 84-year-old woman with hypertension, hyperlipidemia, and anxiety who presented with several weeks of spontaneous nontraumatic bruising, fatigue, and exertional dyspnea. Initial evaluation revealed severe normocytic anemia, isolated aPTT prolongation, lack of correction on a mixing study, factor VIII activity <5%, and a high-titer factor VIII inhibitor of 470 Bethesda Units. Lupus anticoagulant testing was initially positive, while anticardiolipin and anti-β2-glycoprotein I antibodies were negative. The patient developed a right upper extremity hematoma and required a packed red blood cell transfusion. Immunosuppressive therapy with high-dose prednisone and weekly rituximab was initiated, with subsequent clinical improvement, normalization of aPTT, recovery of factor VIII activity, and disappearance of the inhibitor during follow-up. This case highlights the importance of considering acquired hemophilia A in elderly patients with spontaneous bleeding and isolated aPTT prolongation, even in the presence of transient lupus anticoagulant positivity.

## Introduction

Idiopathic factor VIII inhibitor, also known as acquired hemophilia A (AHA), is a rare, potentially life-threatening bleeding disorder [1]. This condition is characterized by the development of autoantibodies against factor VIII. In a healthy coagulation pathway, this factor serves as a cofactor for factor IXa; however, its inhibition results in insufficient thrombin generation on the platelet surface, leading to clot destabilization and severe clinical hemorrhage [2].

Approximately 1.5 cases per million people have been reported annually. However, in contrast to congenital hemophilia, the condition affects both sexes equally, with most of the cases presenting in older adults and the elderly, with a median age of 70 [3]. The bleeding phenotype of AHA is variable, ranging from life-threatening bleeds to mild or no bleeding, with subcutaneous hematomas being characteristic of the disease [4]. Furthermore, this condition has a diverse etiology, with almost 50% of cases associated with underlying conditions, including autoimmune disorders (e.g., rheumatoid arthritis or systemic lupus erythematosus), malignancies (both solid and hematologic tumors), dermatological conditions (e.g., psoriasis, pemphigus vulgaris), and rare cases reported during pregnancy. A substantial proportion of cases remain without an identifiable underlying trigger even after a thorough clinical evaluation and are classified as idiopathic [5].

Patients typically present with mucocutaneous bleeding, extensive ecchymosis, or deep tissue hematomas [6]. However, in AHA, hemarthrosis is less likely, which is a major sign of congenital disease. Patients presenting with new-onset spontaneous bleeding clinically and no personal or family history of coagulopathy should be suspected of having AHA.

Clinically, proper diagnosis follows a stepwise framework: beginning with identification of a normal PT/INR alongside an isolated prolonged activated partial thromboplastin time (aPTT). Initial analysis is followed by a mixing study, which suggests the presence of an inhibitor rather than a simple efficacy if failure of aPTT correction is noted. Ultimately, broad suspicion is narrowed by finding low factor VIII activity and finally confirmed and quantified using the Bethesda assay or the Nijmegen-modified Bethesda assay, which serves as the current gold standard for its improved specificity [7-9].

This case report provides details on the diagnostic challenges of a rare case of idiopathic factor VIII inhibitor coagulopathy with a complicated clinical picture due to the presence of multiple autoimmune markers initially in an elderly female patient presenting with severe bleeding. The challenging part included the patient's profound factor VIII suppression and high inhibitor titer, which occurred in the absence of any provocative underlying pathology. Hence, this lack of any identifiable trigger (despite a thorough workup for malignancy and systemic autoimmune disease) leads to the classification of this case as idiopathic, where the unpredictable nature of immune dysregulation is noted in the patient. A combination of high-dose prednisone and rituximab, following gold-standard clinical practices, was utilized to establish and restore hemostatic stability.

## Case presentation

Patient presentation

This is the case of an 84-year-old female with a past medical history of hypertension, hyperlipidemia, and anxiety who presents to the emergency room with spontaneous easy bruising. The patient endorses nontraumatic bruising to the bilateral upper extremities and back, which first appeared several weeks ago. She has also had fatigue and shortness of breath on exertion during this time. Denies active bleeding, hematochezia, hematemesis, fever, chills, weight loss, or night sweats. She denies a history of trauma or recent falls, and she is not on antiplatelet or anticoagulation therapy.

Physical examination

On admission, vitals were Temp (98.7F), BP (143/68), HR (70/min), RR (16/min), and SpO2 (98%) on ambient air. Physical exam remarkable for pale skin with widespread ecchymoses to the upper back, arms, and chest. There is bilateral lower extremity pitting edema.

Diagnostic assessment and investigations

The diagnostic assessment began with initial labs showing WBC (11.3), hemoglobin (7.4), mean corpuscular volume (91), and platelets (275,000). Furthermore, coagulation studies revealed an isolated, markedly prolonged aPTT of 121 seconds with a normal PT of 13.1 and an INR of 1.18. We followed a stepwise diagnostic framework, failing to correct the mixing study. At this stage, the differential diagnosis remained broad (Table 1).

**Table 1:** Differential diagnosis aPTT: Activated Partial Thromboplastin Time, PT: Prothrombin Time, INR: International Normalized Ratio, AHA: Acquired Hemophilia A, BU: Bethesda Units

Differential Diagnosis	Distinguishing Features	How it was Ruled Out/In
Acquired Hemophilia A (Factor VIII Inhibitor)	Isolated prolonged aPTT, no correction on mixing, bleeding out of proportion to lab values, often in elderly.	Confirmed: Factor VIII level <5% and Factor VIII inhibitor level 470 BU.
Lupus Anticoagulant (Antiphospholipid Syndrome)	May prolong aPTT and may fail to correct on mixing studies; however, it is usually associated with thrombosis rather than spontaneous soft tissue bleeding.	Considered because lupus anticoagulant was initially positive. However, the patient had a bleeding phenotype, anticardiolipin and anti-β2-glycoprotein I antibodies were negative, and repeat lupus anticoagulant testing became negative. Findings favored acquired hemophilia A rather than antiphospholipid syndrome.
Acquired Factor IX, XI, or XII Inhibitor	Isolated factor deficiency on aPTT-based assay; Factor XII deficiency is not associated with bleeding.	Ruled Out: Specific factor assays confirmed Factor VIII deficiency.
Coagulation Factor Deficiency (e.g., Liver Disease, Vitamin K Deficiency)	Usually affects multiple factors (PT and PTT), corrects on mixing study.	Ruled Out: Normal PT/INR, and PTT did not correct on mixing.
Medication-Induced (e.g., new anticoagulant)	History of anticoagulant use (apixaban, rivaroxaban, heparin) which can elevate PTT.	Ruled Out: Patient denied use of antiplatelet or anticoagulant therapy.

Subsequent factor assay confirmed the factor VIII level measured was <5%, and this diagnosis was finalized within the first 48 hours of admission, with a Bethesda assay showing a high-titer factor VIII inhibitor at 470 Bethesda Units (BU). However, our patient did not meet the criteria for antiphospholipid syndrome (APS). An antiphospholipid panel was performed to differentiate between a clotting inhibitor (lupus anticoagulant) and a bleeding inhibitor (factor VIII), as both can cause a non-correcting prolonged aPTT. Labs were also notable for lupus anticoagulant positivity with confirmation on hexagonal phase and negative anti-β2-glycoprotein I antibody and anticardiolipin (Table 2). 

**Table 2:** Initial laboratory data WBC: White Blood cells, MCV: Mean Corpuscular Volume, PT: Prothrombin time, INR: International Normalized Ratio, PTT: Partial Thromboplastin Time

Laboratory Data	Result	Reference Range
WBC	11.3	4.0-10.0 K/µL
Hemoglobin	7.4	12.0-16.0 g/dL
MCV	91	80-100 fL
Platelets	275,000	150,000-450,000 /µL
PT	13.1	11.0-13.5 sec
INR	1.18	0.9-1.1
PTT	121	25-35 sec
Mixing Study (PTT)	No correction	Correction indicates factor deficiency
Factor VIII Level	<5%	50-150%
Factor VIII Inhibitor	470	<0.5 Bethesda Units (BU)
Lupus Anticoagulant	Positive	Negative
Anticardiolipin Antibody	Negative	Negative
Beta-2 Glycoprotein	Negative	Negative

No clear secondary cause was identified after clinical evaluation. The patient had no known active malignancy, autoimmune disease, recent pregnancy, dermatologic disorder, or exposure to antiplatelet or anticoagulant therapy. Therefore, after exclusion of clinically evident associated conditions, the case was classified as idiopathic acquired hemophilia A.

Treatment and hospital course

The patient's hospital course was complicated shortly after administration due to the development of new swelling of the right upper extremity, with an ultrasound demonstrating a hematoma. This local progression, along with a drop in hemoglobin, necessitated the transfusion of 2 units of packed red blood cells. Given the diagnosis of an acquired factor VIII inhibitor with severe bleeding and a high inhibitor titer, immunosuppressive therapy was initiated immediately. The patient was initiated on prednisone 80 mg daily with PCP prophylaxis (trimethoprim-sulfamethoxazole) and GI prophylaxis (pantoprazole). Rituximab was initiated weekly for a total of four doses to target immune tolerance and eradicate the inhibitor. While bypassing agents were considered for hemostatic control, they were ultimately withheld due to the patient’s advanced age and cardiovascular comorbidities, favoring a conservative approach as the bleeding remained localized. 

Outcome and follow-up

The patient showed significant clinical improvement with resolution of bleeding symptoms and was discharged home in stable condition. On her first follow-up visit, she remained clinically stable with no new bleeding episodes. Interval laboratory testing demonstrated significant improvement: her PTT had normalized from 121 seconds on admission to 26 seconds, and her Factor VIII level had risen from less than 5% to 26%. The Factor VIII inhibitor titer, which was initially 470 Bethesda units, improved to 106 Bethesda units and was subsequently not measurable by the lab once her Factor VIII level reached 26 units. The previously positive lupus anticoagulant screen had also converted to negative. The patient had completed four weekly doses of Rituximab for immune tolerance induction and was continued on a prednisone taper, along with trimethoprim-sulfamethoxazole and pantoprazole.

At her subsequent follow-up, the patient continued to do well with no bleeding concerns. Her PTT remained normal at 28 seconds, and her Factor VIII level had normalized to 121%. The factor VIII inhibitor remained undetectable. Given this excellent response, the prednisone was discontinued along with trimethoprim-sulfamethoxazole and pantoprazole. She will continue physical and occupational therapy at home and follow up in the hematology clinic for regular surveillance.

## Discussion

**Figure 1:**
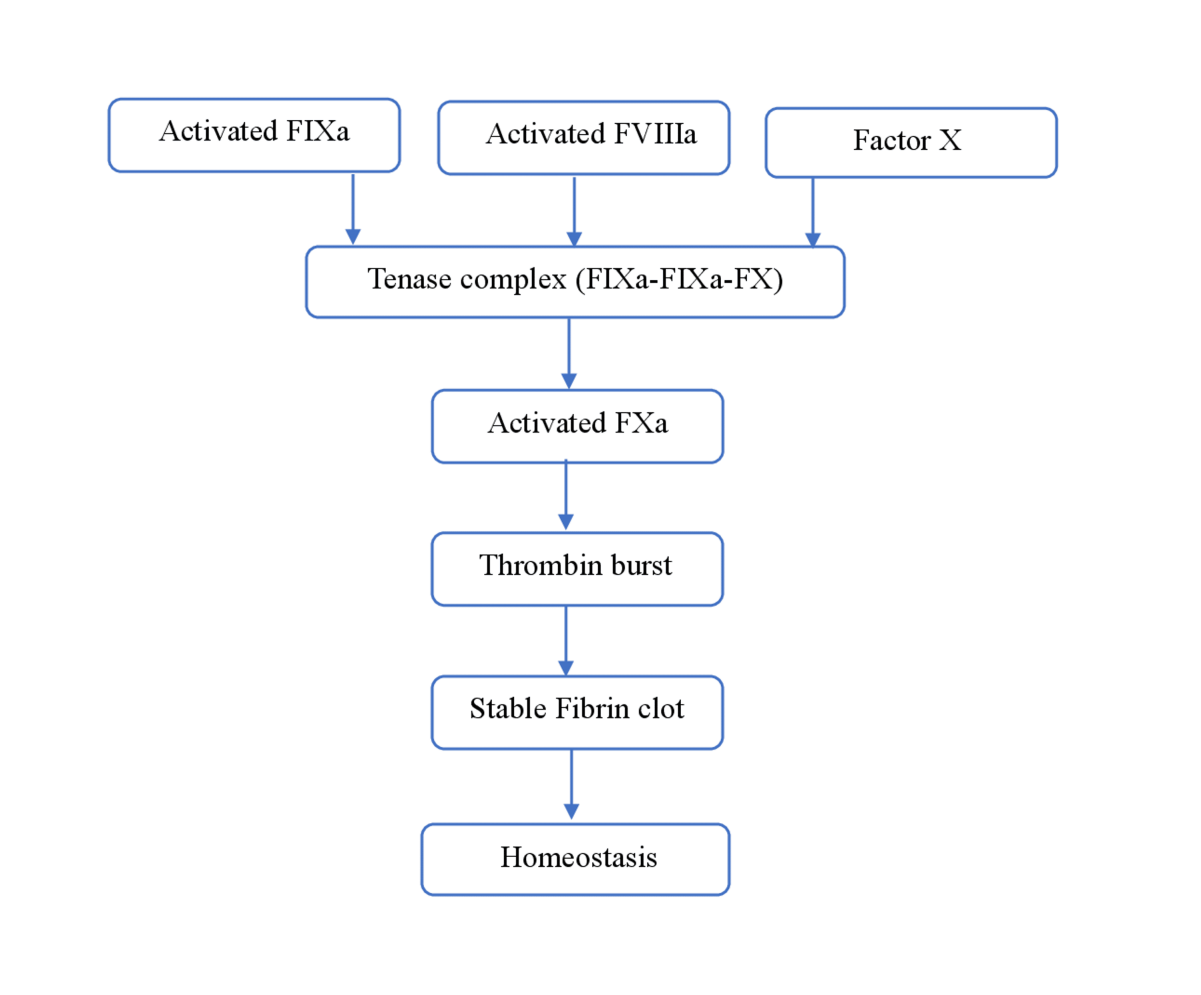
Normal hemostasis and the intrinsic tenase complex A flowchart illustrating the mechanism of normal homeostasis, created using Microsoft Word, showing neutralizing autoantibodies that disrupt hemostasis by binding to the functional domains of factor VIII, causing steric hindrance that prevents its interaction with factor IXa and platelet phospholipids. This blockade inhibits the assembly of the intrinsic tenase complex, leading to a failure of the thrombin burst and resulting in the formation of unstable fibrin clots and severe clinical hemorrhage.

**Figure 2:**
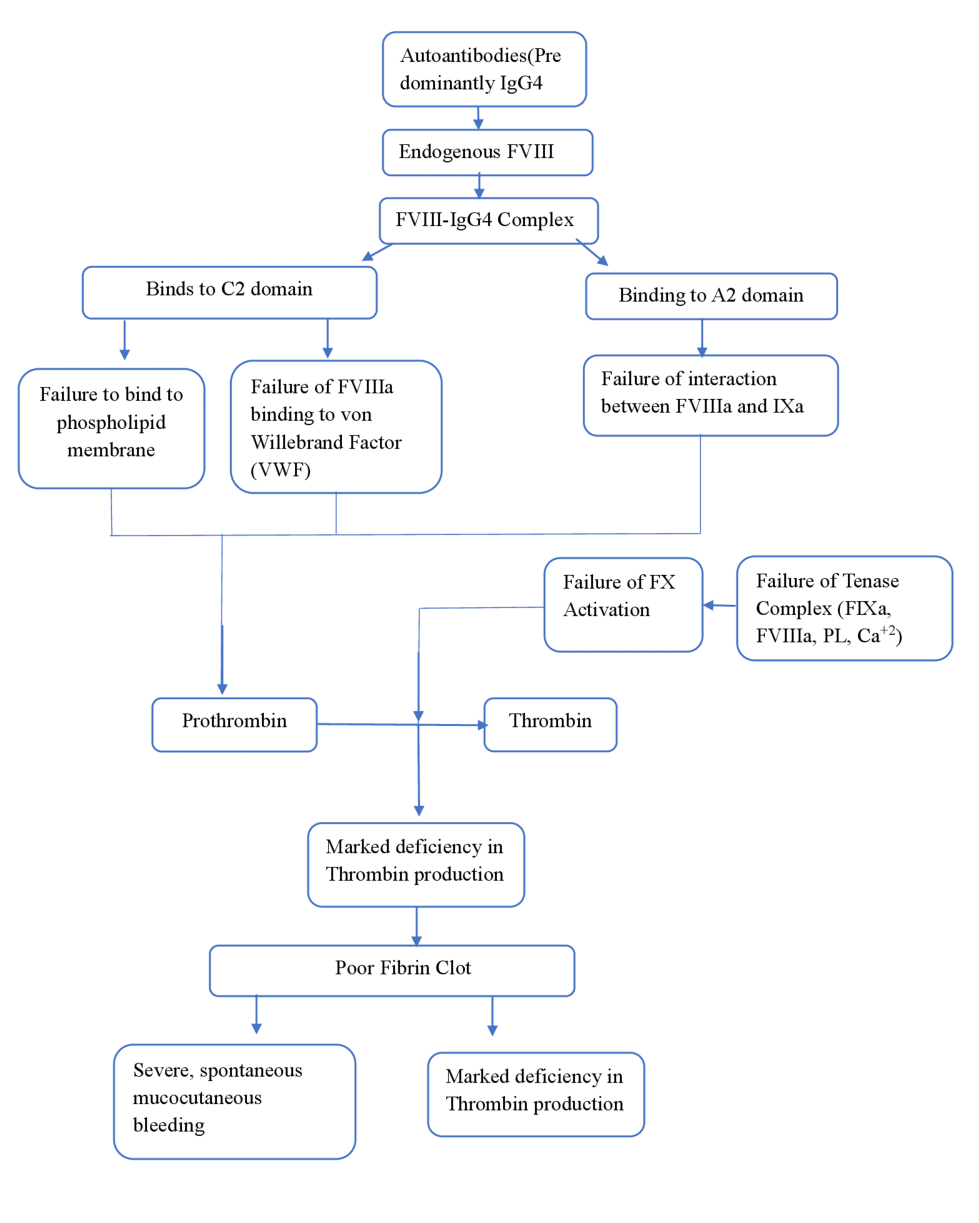
Pathophysiology of acquired factor VIII inhibition Flowchart illustrating pathophysiology of acquired factor VIII inhibition, created using Microsoft Word. This deficiency is driven by neutralizing autoantibodies (predominantly IgG4) that target the A2 and C2 domains of the factor VIII molecule. This binding causes steric hindrance, preventing factor VIII from interacting with factor IXa, von Willebrand factor, and the platelet phospholipid membrane. Consequently, the intrinsic tenase complex fails to assemble, resulting in a marked deficiency of thrombin production and the formation of unstable fibrin clots, leading to severe, spontaneous hemorrhage.

An acquired factor VIII inhibitor is a rare condition with its pathophysiology centered on the coagulation cascade. Factor VIII acts as a critical factor for the activation of factor IX to IXa, which further results in the formation of a complex. This complex activates factor X, facilitating thrombin formation on an activated platelet. The autoantibodies, which are predominantly of the IgG4 subclass, are directed against specific functional domains of the factor VIII protein, leading to its neutralization or accelerated clearance from the circulation [10]. As described by Alice D. et al., the inhibitor binds to sites on the factor VIII molecule that are critical for its procoagulant function, such as the A2, A3, and C2 domains, thereby interfering with the tenase complex assembly [11]. The presence of inhibitors leads to a profound failure of thrombin generation on activated platelets, manifesting as severe, often spontaneous, mucocutaneous or soft tissue bleeding [12] (Figures 1, 2).

Autoantibodies, particularly lupus anticoagulants, along with the factor VIII inhibitor, indicate a broad state of immune dysregulation in this patient. The case diagnosis was confirmed after performing mixing studies when the coagulation cascade failed to correct, and a prolonged aPTT of 121 seconds was noted, pointing towards an inhibitor rather than a simple factor deficiency [8,13]. A diagnosis of AHA should be considered in the differential for a patient presenting with acute bleeding and an unexplained prolonged aPTT [4].

A two-fold treatment strategy was incorporated for this patient to achieve hemostasis and eradicate the inhibitor. A markedly high titer of inhibitor was reported in the patient, necessitating immunosuppression as the cornerstone in her care [14]. The patient was initiated on high-dose prednisone (80 mg) and rituximab in an effort to downregulate autoantibody production. According to international recommendations, immunosuppressive therapy for autoantibody eradication should be initiated promptly in all patients with AHA, with regimens typically composed of corticosteroids, cyclophosphamide, or rituximab [4]. Prophylaxis is required with the administration of high-dose steroids, and the patient was appropriately placed on trimethoprim-sulfamethoxazole for PCP prophylaxis and pantoprazole for GI protection. The prognosis is unpredictable, particularly in the elderly, due to fatal hemorrhage or infection during treatment. The affected population often has underlying comorbid conditions, necessitating the use of antiplatelets or anticoagulation medication, which can contribute to severe life-threatening bleeds and require emergency reversal agents. However, the dual approach with this patient and her improvement, along with discharge, supports a positive initial response, though long-term monitoring is necessary for inhibitor relapse caution [14].

## Conclusions

Acquired hemophilia A should be suspected in older adults presenting with spontaneous soft tissue or mucocutaneous bleeding and isolated aPTT prolongation, particularly when mixing studies fail to correct it. Confirmation requires measurement of factor VIII activity and inhibitor quantification using the Bethesda or Nijmegen-modified Bethesda assay. Early recognition is essential to guide hemostatic support, initiate immunosuppressive therapy, and reduce the risk of severe hemorrhagic complications. This case also illustrates that transient lupus anticoagulant positivity may coexist with acquired factor VIII inhibitors and should be interpreted cautiously within the clinical context. Long-term surveillance of factor VIII activity remains necessary because relapse can occur after apparent remission.
